# GLP-1 and GIP Changes after Sleeve Gastrectomy and Weight Regain in Adolescents. Do we need a Boost?

**DOI:** 10.1007/s11695-025-08168-x

**Published:** 2025-09-01

**Authors:** Mohamed Shehata, Ahmed Elhaddad, Mohamed Mansour, Sherif Shehata, Ashraf El Attar

**Affiliations:** Tanta University, Tanta, Egypt

**Keywords:** Adolescent obesity, Sleeve gastrectomy, Weight regain, GLP-1, GIP, Semaglutide

## Abstract

**Purpose:**

Sleeve gastrectomy (LSG) is effective, but weight regain (WR) and variable hormonal responses challenge long-term outcomes. This study evaluated long-term weight and incretin trajectories after LSG in adolescents and assessed the impact of adjunct semaglutide therapy for patients with WR.

**Materials and Methods:**

A retrospective cohort of 264 adolescents (mean age 15 ± 3 years; 74% female) underwent standardized LSG and was followed for five years with annual measurements of weight, BMI, %TWL, %EWL, GLP-1 and GIP. WR was defined as ≥ 10% gain from nadir plus < 50% excess weight loss at 18 months. Patients meeting these criteria (n = 62) received semaglutide from postoperative year 3.

**Results:**

Mean weight dropped from 133 to 87 kg by year 2, with %EWL peaking at 68% and declining to 63% by year 5. GLP-1 and GIP levels rose in the first postoperative year and diminished thereafter. Adolescents with WR exhibited more pronounced attenuation of incretin responses and larger gastric volumes than those without WR. Semaglutide increased mean %EWL in WR patients from 34 to 68% by year 3. Metabolic comorbidities improved across the cohort.

**Conclusions:**

LSG produces sustained weight loss and metabolic benefits in adolescents. Incretin surges attenuate over time, particularly among those experiencing WR. A one-year course of semaglutide partially reversed WR, suggesting a potential adjunctive role for GLP-1 agonists in selected patients. Early hormonal differences warrant prospective research to clarify predictive value for weight regain.

## Introduction

Adolescent obesity poses long-term health risks and is increasingly prevalent worldwide [1, 2]. Traditional interventions often fail to produce durable results, leading to growing interest in bariatric surgery (BS) [3], particularly laparoscopic sleeve gastrectomy (LSG), which is now the most commonly performed procedure in this age group. Despite its effectiveness, concerns persist about long-term outcomes, including weight regain (WR), particularly due to developmental, behavioral, and hormonal factors unique to adolescents [4, 5].

Adolescents are a unique population due to their physiological and psychological development. Pubertal hormonal changes, ongoing linear growth, and evolving eating behaviors may influence weight loss outcomes and the risk of weight regain (WR). Moreover, early-onset obesity has long-term implications, increasing the likelihood of adult obesity and associated metabolic diseases [6, 7].

LSG’s effects extend beyond mechanical restriction, involving significant changes in gastric emptying and gut hormones, particularly glucagon-like peptide-1 (GLP-1) and glucose-dependent insulinotropic peptide (GIP) [8]. While GLP-1 has been extensively studied for glucose control and appetite regulation, the role of GIP remains less defined. Post-LSG, early surges in these hormones contribute to weight loss and improved insulin sensitivity, though this effect may wane over time [9, 10].

While LSG has proven to be a highly effective intervention for adolescent obesity, offering significant weight reduction and resolution of associated comorbidities, recent long-term studies further solidify its role. For instance, a comprehensive seven-year follow-up study by Shehata et al. (2025) demonstrated sustained weight loss and remarkable improvements in metabolic health among adolescents undergoing LSG, reinforcing its durability as a therapeutic option [11].

WR, typically emerging 2–3 years postoperatively, is associated with declining incretin responses and potential anatomic or behavioral adaptations. Pharmacological agents like semaglutide, a GLP-1 receptor agonist, offer potential adjunctive strategies to mitigate WR [12, 13].

The growing body of evidence examining gut hormone physiology and effects in patients with obesity following sleeve gastrectomy (SG) highlights the critical role these hormones play in both the mechanisms and management of obesity [13].

This study aims to evaluate adolescents who underwent LSG at our center by assessing body mass index (BMI), percent excess weight loss (%EWL), pre- and post-operative GLP-1 and GIP levels, WR incidence, and the impact of a Semaglutide boost on WL.

## Materials and Methods

### Patients and Study Design

This retrospective cohort study **included** adolescents aged 10–19 years with morbid obesity who underwent LSG in the form of antral resection [the first staple firing started 2 cm from the gastro-duodenal junction (pyloric ring)] using 36-French bougie [10], identified from institutional electronic medical records at Tanta University Hospitals and its affiliated hospitals, Tanta, Egypt, between January 2016 and December 2019.

**Exclusion criteria** were patients aged < 10 or > 19 years and those who underwent LSG with antral preservation [the first staple firing started more than 2 cm from the pyloric ring], alternative bariatric procedures, such as Roux-en-Y gastric bypass (RYGB) or laparoscopic adjustable gastric banding (LAGB) during the study period.

### Ethical Issues

The study was conducted following the Declaration of Helsinki and approved by the Institutional and Regional Ethical Committees of Faculty of Medicine, Tanta University (reference number: 36264PR1087/2/25) and had Clinical trial registration from Pan African Clinical Trial Registry (PACTR202504698770932). Written informed consent was obtained from all patients and their guardians. A separate informed consent process was conducted for those administered Semaglutide, as the intervention was not a part of standard postoperative care.

### Preoperative Assessment and Behavioral Screening

Eligibility required documented failure of at least 6 months of structured medical weight management under a multidisciplinary bariatric team. All patients underwent comprehensive preoperative psychological evaluation by a pediatric psychologist to assess readiness, compliance, and presence of eating disorders or untreated mood disturbances. Structured counseling sessions with parents were conducted to ensure psychosocial preparedness and adherence to postoperative protocols.

### Dietary and Lifestyle Support

Patients received standardized nutritional counseling before and after surgery from certified dietitians. Postoperative dietary plans were implemented in stages (clear fluids, full liquids, pureed foods, then soft and solid foods) over 6 weeks. Patients also attended group sessions and individual follow-ups for behavioral reinforcement, physical activity encouragement, and vitamin supplementation compliance throughout the follow-up period.

### Data Collection

A dedicated bariatric follow-up clinic coordinated annual visits for all participants. Families were contacted via telephone and email reminders, and follow-up appointments were scheduled to coincide with routine clinical care whenever possible. These measures resulted in a high retention rate: 264 of the 293 enrolled adolescents (90.1%) completed the full five-year follow-up. For each annual assessment, anthropometric measures and blood samples were obtained from all attendees, and there were no missing hormone measurements at the scheduled time points.

Clinical, demographic, and metabolic data were extracted from electronic medical records and patient charts, including preoperative and postoperative body mass index (BMI), percent total weight loss (%TWL), percent excess weight loss (%EWL), gastric volume, HbA1c, GLP-1, and GIP levels. Operative details (e.g., procedure duration, hospital stay) and comorbidity profiles (diabetes, hypertension, and dyslipidemia) were documented. Gastric volume was assessed annually in the fasting state using standardized abdominal MRI, segmentation software was used to reconstruct the gastric sleeve in 3D, yielding the remnant gastric volume in milliliters.

Remission of type 2 diabetes mellitus (T2DM) was defined as HbA1c < 6.5% without the use of anti-diabetic medications, in accordance with the American Diabetes Association (ADA) criteria [14]. Hypertension remission was defined as systolic and diastolic blood pressure < 130/80 mmHg without antihypertensive therapy, consistent with pediatric hypertension guidelines [15]. Dyslipidemia remission was defined as normalization of LDL (< 130 mg/dL), HDL (> 40 mg/dL), and triglycerides (< 150 mg/dL) without lipid-lowering medications, per the National Lipid Association recommendations [16].

### Hormonal Assays

Blood samples for GLP-1 and GIP were collected at baseline and annually postoperatively at fasting and at 30, 60, and 120 min after a standardized mixed meal. All patients fasted overnight for at least 10 h. A 300 kcal standardized liquid meal (Ensure Plus®; 57% carbohydrate, 15% protein, 28% fat) was consumed within 10 min. Blood sampling was performed at 0 (fasting), 30, 60, and 120 min post-ingestion. All tests were conducted between 8:00 and 9:00 AM to minimize diurnal variability. At each time point, anthropometric data and blood samples were collected for all 264 patients. All samples were processed immediately after collection, centrifuged, and stored at − 80°C. Hormone levels were quantified using commercially available enzyme-linked immunosorbent assay (ELISA) kits [10].

GLP-1 and GIP levels were quantified using validated commercial sandwich ELISA kits (BioVendor®, Czech Republic) designed for human plasma, with all samples analyzed in duplicate according to the manufacturer’s instructions.

For patients receiving semaglutide, hormonal measurements were performed without discontinuing the medication, as per standard clinical follow-up (only one sample during semaglutide intake). These patients remained on active treatment during the time of sampling, allowing real-world assessment of endogenous GLP-1 and GIP levels under pharmacologic modulation.

### Surgical Procedure

LSG was performed according to standardized protocols at our center. LSG was conducted utilizing a 36-French bougie, maintaining a distance of approximately 2 cm from the pylorus [10]. The procedural details were documented in the electronic medical records.

### Semaglutide Intervention

Semaglutide therapy was initiated at the start of year 3 for adolescents meeting the WR criteria (≥ 10% weight regain from the nadir postoperative weight and %EWL < 50% at 18 months) [17, 18]. Treatment began at 0.25 mg weekly for 4 weeks, escalated to 0.5 mg weekly for another 4 weeks, then maintained at 1.0 mg weekly for the remainder of the 12-month course. After 12 months, Semaglutide was gradually tapered over 3 months by reducing the dose to 0.5 mg for one month and then 0.25 mg weekly for two months before discontinuation. Patients were monitored monthly for glycemic control, adverse effects, and weight progression.

It is important to note that semaglutide administration was initiated only in patients aged 18 years and older, consistent with current regulatory approval for its use in adults.

### Response and Tolerability Criteria

A clinical response was defined as a ≥ 10% increase in %EWL following Semaglutide. Adverse events including gastrointestinal symptoms were documented using standardized checklists. All patients tolerated the treatment well, and no serious adverse effects occurred.

### Follow-up

Postoperative evaluations were conducted annually for five years. Outcomes included longitudinal monitoring of weight changes, gastric volume, hormonal levels (fasting/postprandial GLP-1 and GIP), and comorbidity status. Clinically significant WR was defined as a ≥ 10% increase from nadir weight and < 50% EWL at 18 months. In cases of significant regain, the potential impact of Semaglutide supplementation on WL maintenance was assessed.

### Handling of Missing Data

Out of 293 initial patients, 29 were lost to follow-up. These cases were excluded from the final analysis. No data imputation was used. The results reflect the outcomes of 264 patients with complete follow-up.

### Primary and Secondary Outcomes

The **primary** outcome evaluated longitudinal changes in fasting/postprandial GLP-1 levels post-LSG over five years. **Secondary** outcomes included the descriptive analysis of GIP trajectories, weight regain (WR) patterns, the response to semaglutide intervention in WR patients, reductions in BMI, %TWL, %EWL, gastric volume, and HbA1c; partial WR post-year 2; and comorbidity resolution. While hormone and weight patterns were compared across groups, no formal correlation analysis was performed.

### Sample Size Calculation

The sample size calculation was done by G*Power 3.1.9.2 (Universitat Kiel, Germany). We performed a pilot study, comprising five cases, which demonstrated the mean (± SD) of GLP-1 level was 31.4 ± 9 pmol/L preoperative and 39.4 ± 9.4 pmol/L after 1year. The sample size was based on the following considerations: 0.869 effect size, 95% confidence level, 95% power of the study. Therefore, at least 85 patients were to be recruited in our study. This calculation was based solely on changes in GLP-1; the study was not powered to detect associations between GLP-1/GIP and weight regain or to evaluate the impact of Semaglutide.

### Statistical Analysis

Statistical analysis was done by SPSS v26 (IBM^Inc.^, Chicago, IL, USA). Quantitative parametric data were presented as mean and standard deviation (SD) and were compared by paired T-test or repeated measures ANOVA. Bonferroni correction was applied for multiple comparisons. Qualitative variables were presented as frequency and percentage and were compared using the McNemar test. A two-tailed P < 0.05 was considered statistically significant. No correlation analyses were performed between hormonal changes and weight regain; temporal associations were described descriptively.

## Results

### Study Cohort Characteristics

**Fig. 1:**
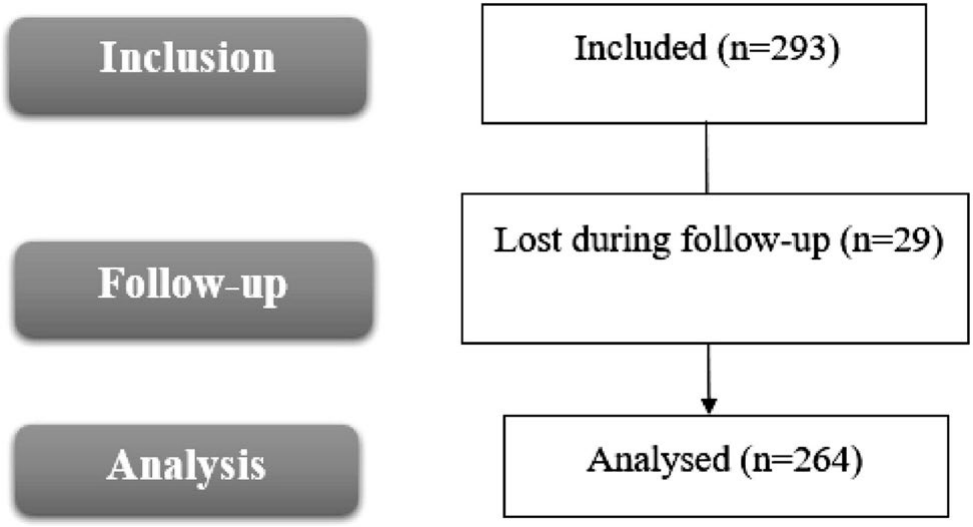
Flow chart of the studied patients

This study assessed 293 patients who underwent LSG with the first staple line 2 cm from the pyloric ring for eligibility. 29 patients were lost in follow-up, 264 (90.1%) patients completed the five-year followe-up and were included in the analyses (Fig. 1).

Complete anthropometric and hormonal data were available for all 264 patients at each time point, and these are the data presented across tables and figures.

The mean age was 15.04 ± 2.99 years. There were 69 males (26.14%) and 195 females (73.86%). Mean weight was 132.8 ± 9.67 kg, Mean height was 166.52 ± 9.15 cm, Mean BMI was 48.38 ± 6.95 kg/m.^2^, operative time was 63.75 ± 11.4 min, and hospital stay was 1.83 ± 0.79 days (Table 1).

**Table 1:** Demographic data, operative time and hospital stay of all the studied patients

Parameter		(*n* = 264)
**Age (years)**		15.04 ± 2.99
**Sex**	**Male (n., %)**	69 (26.14%)
	**Female (n., %)**	195 (73.86%)
**Weight (kg)**		132.8 ± 9.67
**Height (cm)**		166.52 ± 9.15
**BMI (kg/m**^**2**^**)**		48.38 ± 6.95
**Operative time (min)**		63.75 ± 11.4
**Hospital stay (days)**		1.83 ± 0.79

Data are presented as mean ± SD or frequency (%)

### Weight Trajectory and Percent Excess Weight Loss (%EWL)

Weight and BMI significantly declined over the first two years postoperatively, reaching nadir values at year 2, followed by gradual weight regain through year 5. Mean %TWL peaked at 34.97 ± 11.18% in year 2, then decreased to 32.31 ± 8.33% at year 5. Mean %EWL peaked at 68.5 ± 25.51% in year 2, then decreased to 63.3 ± 19.63% at year 5. Gastric volume increased progressively, from 120.8 ± 19.7 mL at year 1 to 219.9 ± 19.04 mL at year 5 (Table 2).

**Table 2:** Weight, BMI, %TWL, %EWL and gastric volume of all the studied patients

(*n* = 264)		*P*-value	Mean difference (95% CI)
Weight (kg)			
Preoperative	132.8 ± 9.67		
1 year	92.3 ± 12.08	** < 0.001**	−40.47 (−42.34: −38.6)
2 years	86.6 ± 17.23	** < 0.001**	−46.21 (−48.6: −43.82)
3 years	87.1 ± 15.83	** < 0.001**	−45.72 (−47.96: −43.48)
4 years	88.6 ± 14.35	** < 0.001**	−44.22 (−46.31: −42.13)
5 years	90.1 ± 14.17	** < 0.001**	−42.73 (−44.81: −40.66)
BMI (kg/m^2^)			
Preoperative	48.4 ± 6.95		
1 year	33.6 ± 5.98	** < 0.001**	−14.75 (−15.86: −13.64)
2 years	31.6 ± 7.74	** < 0.001**	−16.78 (−18.04: −15.52)
3 years	31.8 ± 7.48	** < 0.001**	−16.58 (−17.81: −15.34)
4 years	32.3 ± 7.09	** < 0.001**	−16.03 (−17.23: −14.83)
5 years	32.9 ± 7	** < 0.001**	−15.5 (−16.69: −14.31)
TWL (%)			
1 year	30.63 ± 6.02		
2 years	34.97 ± 11.18	** < 0.001**	4.34 (2.8: 5.87)
3 years	34.61 ± 9.68	** < 0.001**	3.98 (2.6: 5.35)
4 years	33.45 ± 8.36	** < 0.001**	2.82 (1.58: 4.07)
5 years	32.31 ± 8.33	**0.002**	1.68 (0.44: 2.92)
EWL (%)			
1 year	59.9 ± 15.81		
2 years	68.5 ± 25.51	** < 0.001**	8.67 (5.05: 12.3)
3 years	67.9 ± 22.71	** < 0.001**	8.03 (4.68: 11.37)
4 years	65.6 ± 20.09	** < 0.001**	5.72 (2.63: 8.81)
5 years	63.3 ± 19.63	** < 0.001**	3.42 (0.37: 6.46)
Gastric volume (ml)			
Preoperative	1132.7 ± 94.77		
1 year	120.8 ± 19.7	** < 0.001**	−1011.99 (−1023.7: −1000.29)
2 years	176.9 ± 18.62	** < 0.001**	−955.88 (−967.56: −944.2)
3 years	183.9 ± 18.63	** < 0.001**	−948.86 (−960.54: −937.19)
4 years	196.8 ± 19.57	** < 0.001**	−935.92 (−947.62: −924.22)
5 years	219.9 ± 19.04	** < 0.001**	−912.88 (−924.56: −901.19)

Data are presented as mean ± SD. *BMI* Body mass index, *TWL* Total weight loss, *EWL* Excess weight loss, *CI* Confidence interval

### Metabolic Improvements and Comorbidity Resolution

As indicated in Table 3, HbA1c improved significantly from 7.12 ± 0.85% preoperatively to 5.36 ± 0.46% at year 5. There were substantial and statistically significant reductions in comorbidities:

- Type 2 diabetes decreased from 36.36% to 5.68%
- Hypertension from 48.86% to 6.82%
- Dyslipidemia from 43.18% to 4.55%

**Table 3:** HbA1c and comorbidities of all the studied patients

(*n* = 264)			*P* value	MD/RR (95% CI)
HbA1c (%)				
Preoperative		7.12 ± 0.85		
1 year		5.59 ± 0.73	** < 0.001**	−0.53 (−0.66: −0.39)
2 years		5.47 ± 0.55	** < 0.001**	−0.65 (−0.77: −0.53)
3 years		5.45 ± 0.52	** < 0.001**	−0.67 (−0.79: −0.55)
4 years		5.38 ± 0.49	** < 0.001**	−0.74 (−0.86: −0.62)
5 years		5.36 ± 0.46	** < 0.001**	−0.76 (−0.88: −0.64)
Diabetes mellitus (n., %)				
Preoperative	96 (36.36%)			
1 year	42 (15.91%)		** < 0.001**	2.29(1.66:3.15)
2 years	27 (10.23%)		** < 0.001**	3.56(2.4:5.26)
3 years	24 (9.09%)		** < 0.001**	4(2.65:6.05)
4 years	18 (6.82%)		** < 0.001**	5.33(3.32:8.56)
5 years	15 (5.68%)		** < 0.001**	6.4(3.82:10.73)
Hypertension (n., %)				
Preoperative	129 (48.86%)			
1 year	81 (30.68%)		** < 0.001**	1.59(1.28:1.98)
2 years	51 (19.32%)		** < 0.001**	2.53(1.92:3.33)
3 years	42 (15.91%)		** < 0.001**	3.07(2.27:4.16)
4 years	24 (9.09%)		** < 0.001**	5.38(3.6:8.03)
5 years	18 (6.82%)		** < 0.001**	7.17(4.51:11.38)
Dyslipidemia (n., %)				
Preoperative	114 (43.18%)			
1 year	63 (23.86%)		** < 0.001**	1.81(1.4:2.34)
2 years	42 (15.91%)		** < 0.001**	2.71(1.99:3.7)
3 years	30 (11.36%)		** < 0.001**	3.8(2.64:5.47)
4 years	21 (7.95%)		** < 0.001**	5.43(3.52:8.37)
5 years	12 (4.55%)		** < 0.001**	9.5(5.37:16.8)

Data are presented as mean ± SD or frequency (%). *MD* Mean difference, *RR* Risk ratio, *CI* Confidence interval

### GLP-1 and GIP Hormonal Responses

#### Overall Cohort

**Fig. 2:**
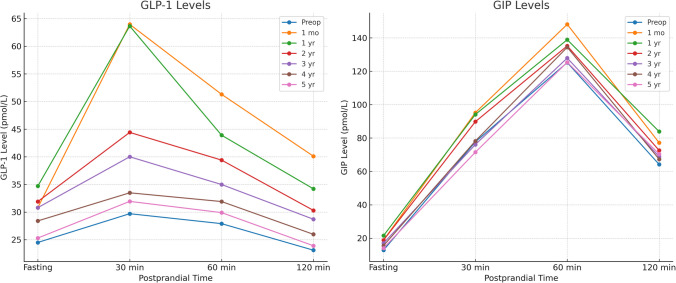
GLP-1 and GIP levels of all the studied patients at all time points

**Fig. 3:**
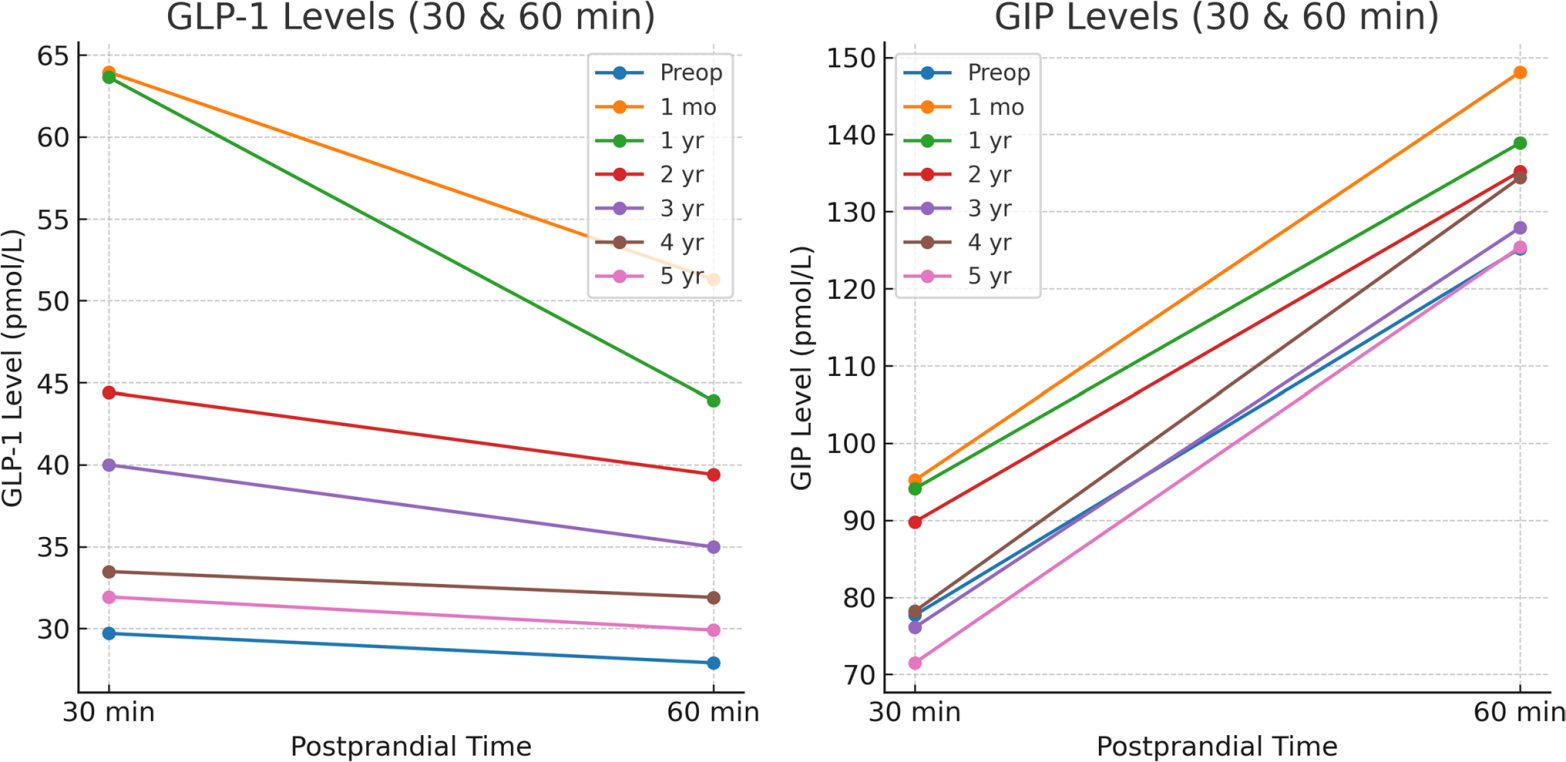
GLP-1 and GIP levels at 30 and 60 min postprandial of all the studied patients

Table 4 illustrates the longitudinal changes in GLP-1 and GIP levels across fasting and postprandial time points in the overall cohort. Both hormones exhibited a marked postoperative surge, with the highest levels observed during the first postoperative year, particularly at 30 and 60 min postprandial. This initial enhancement gradually diminished over time, with a near return to preoperative levels by year five. Notably, GLP-1 responses remained statistically significant through year four, with marginal changes by year five, whereas GIP levels showed sustained elevation up to year three with progressive attenuation thereafter (Figs. 2 and 3).

**Table 4:** GLP-1 and GIP levels of all the studied patients

(*n*=264)	Fasting	30 min	60 min	120 min
GLP-1 level (pmol/L)				
Preoperative	24.5 ± 9.18	29.7 ± 11.61	27.9 ± 11.57	23.1 ± 7.9
1 month	30.8 ± 9.31	63.96 ± 14.02	51.3 ± 12.31	40.1 ± 8.95
*P* value	**<0.001**	**<0.001**	**<0.001**	**<0.001**
Mean difference (95% CI)	6.27 (4.69 : 7.85)	34.24 (32.04 : 36.44)	23.31 (21.26 : 25.35)	16.98 (15.54 : 18.43)
1 year	34.7 ± 9.06	63.65 ± 13.9	43.9 ± 12.57	34.2 ± 9.34
*P* value	**<0.001**	**<0.001**	**<0.001**	**<0.001**
Mean difference (95% CI)	10.17 (8.62 : 11.73)	33.93 (31.74 : 36.12)	16 (13.93 : 18.07)	11.04 (9.56 : 12.52)
2 years	31.9 ± 9.02	44.41 ± 14.4	39.4 ± 12.96	30.3 ± 9.61
*P* value	**<0.001**	**<0.001**	**<0.001**	**<0.001**
Mean difference (95% CI)	7.45 (5.89 : 9.01)	14.69 (12.45 : 16.93)	11.47 (9.37 : 13.57)	7.19 (5.69 : 8.7)
3 years	30.8 ± 9.41	40 ± 14.33	34.98 ± 12.88	28.7 ± 9.66
*P* value	**<0.001**	**<0.001**	**<0.001**	**<0.001**
Mean difference (95% CI)	6.35 (4.76 : 7.94)	10.26 (8.03 : 12.49)	7.03 (4.94 : 9.13)	5.55 (4.04 : 7.05)
4 years	28.4 ± 9.09	33.48 ± 13.67	31.9 ± 12.14	25.97 ± 9.15
*P* value	**<0.001**	**<0.001**	**<0.001**	**<0.001**
Mean difference (95% CI)	3.89 (2.32 : 5.45)	3.76 (1.59 : 5.93)	3.99 (1.96 : 6.02)	2.84 (1.38 : 4.3)
5 years	25.3 ± 9.43	31.93 ± 13.61	29.9 ± 11.81	23.9 ± 8.31
*P* value	0.052	**<0.001**	**<0.001**	0.053
Mean difference (95% CI)	0.84 (−0.75 : 2.43)	2.21 (0.05 : 4.38)	1.99 (−0.01 : 3.99)	0.77 (−0.61 : 2.16)
GIP level (pmol/L)				
Preoperative	12.8 ± 4.27	77.7 ± 20.37	125.2 ± 23.17	64.2 ± 17.71
1 month	18.8 ± 5.31	95.2 ± 21.16	148.1 ± 24.39	77.2 ± 18.13
*P* value	**<0.001**	<0.001	**<0.001**	**<0.001**
Mean difference (95% CI)	6.01 (5.18 : 6.83)	17.45 (13.9 : 21.01)	22.97 (18.9 : 27.04)	13.03 (9.97 : 16.09)
1 year	21.5 ± 4.85	94.1 ± 20.41	138.9 ± 22.54	83.9 ± 17.35
*P* value	**<0.001**	<0.001	**<0.001**	**<0.001**
Mean difference (95% CI)	8.65 (7.87 : 9.43)	16.35 (12.86 : 19.84)	13.77 (9.86 : 17.68)	19.73 (16.73 : 22.73)
2 years	18.8 ± 4.84	89.8 ± 20.79	135.2 ± 23.03	72.6 ± 17.86
*P* value	**<0.001**	<0.001	**<0.001**	**<0.001**
Mean difference (95% CI)	5.99 (5.21 : 6.77)	12.07 (8.55 : 15.59)	10.05 (6.1 : 14)	8.43 (5.39 : 11.47)
3 years	16.98 ± 4.67	76.1 ± 20.73	127.9 ± 23.6	68.9 ± 16.67
*P* value	**<0.001**	0.002	**<0.001**	**<0.001**
Mean difference (95% CI)	4.16 (3.4 : 4.93)	−1.69 (−5.2 : 1.83)	2.76 (−1.24 : 6.76)	4.76 (1.82 : 7.7)
4 years	15.5 ± 4.92	78.2 ± 20.13	134.4 ± 24.14	67.3 ± 16.94
*P* value	**<0.001**	0.464	**<0.001**	**<0.001**
Mean difference (95% CI)	2.67 (1.88 : 3.46)	0.41 (−3.05 : 3.88)	9.28 (5.23 : 13.33)	3.11 (0.15 : 6.08)
5 years	14 ± 4.82	71.5 ± 20.04	125.4 ± 24.63	70.4 ± 16.96
*P* value	**<0.001**	<0.001	0.693	**<0.001**
Mean difference (95% CI)	1.2 (0.42 : 1.98)	−6.27 (−9.72 : −2.81)	0.27 (−3.82 : 4.35)	6.23 (3.26 : 9.19)

Data are presented as mean ± SD. *CI* Confidence interval

Statistical comparisons were performed for each postoperative time point versus baseline to assess significance of hormonal changes. Time points with *p* < 0.05 and corresponding 95% CI indicate significant change

No formal correlation analyses (Pearson or Spearman) were conducted between GLP-1 or GIP levels and WR. Instead, group-level comparisons of hormone levels in patients with and without WR were presented descriptively over time to illustrate temporal associations.

### Incidence and Characteristics of the Clinically Significant Weight Regain (WR)

**Fig. 4:**
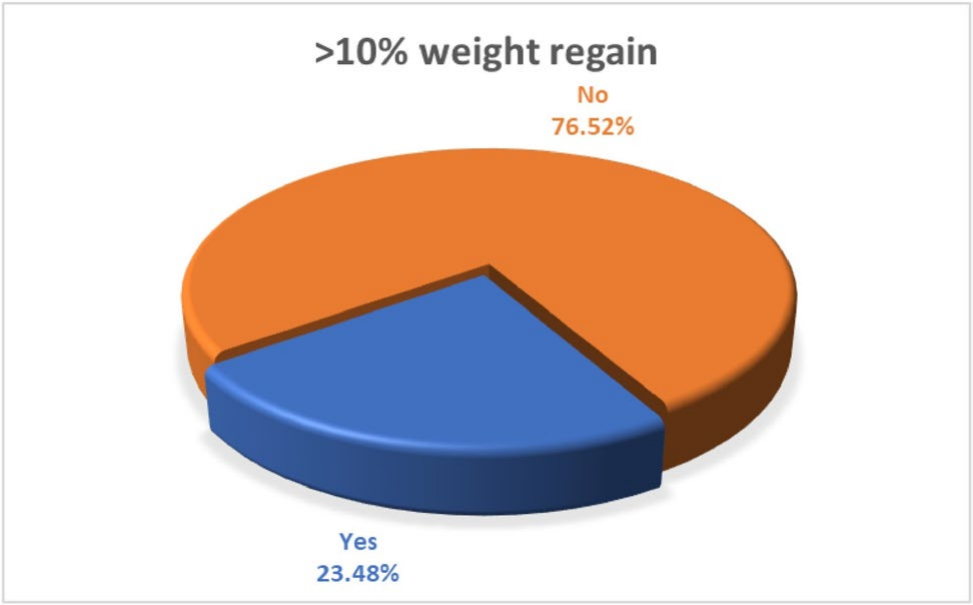
Incidence of clinically significant weight regain among the studied patients

There is normal weight rebound 5–10% starting 2–3 years postoperatively [19]. WL failure is to lose < 50% by 18 months postoperatively [20]. WR is > 10% WR after the 18 months postoperatively [19]. Out of 264 patients 62 patients had clinically significant WR was (≥ 10% increase from nadir weight and < 50% EWL at 18 months) (23.48%) (Fig. 4).

**Fig. 5:**
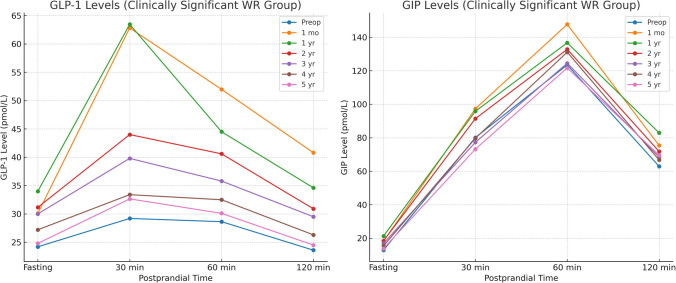
GLP-1 and GIP levels in the clinically significant weight regain group at all time points

**Fig. 6:**
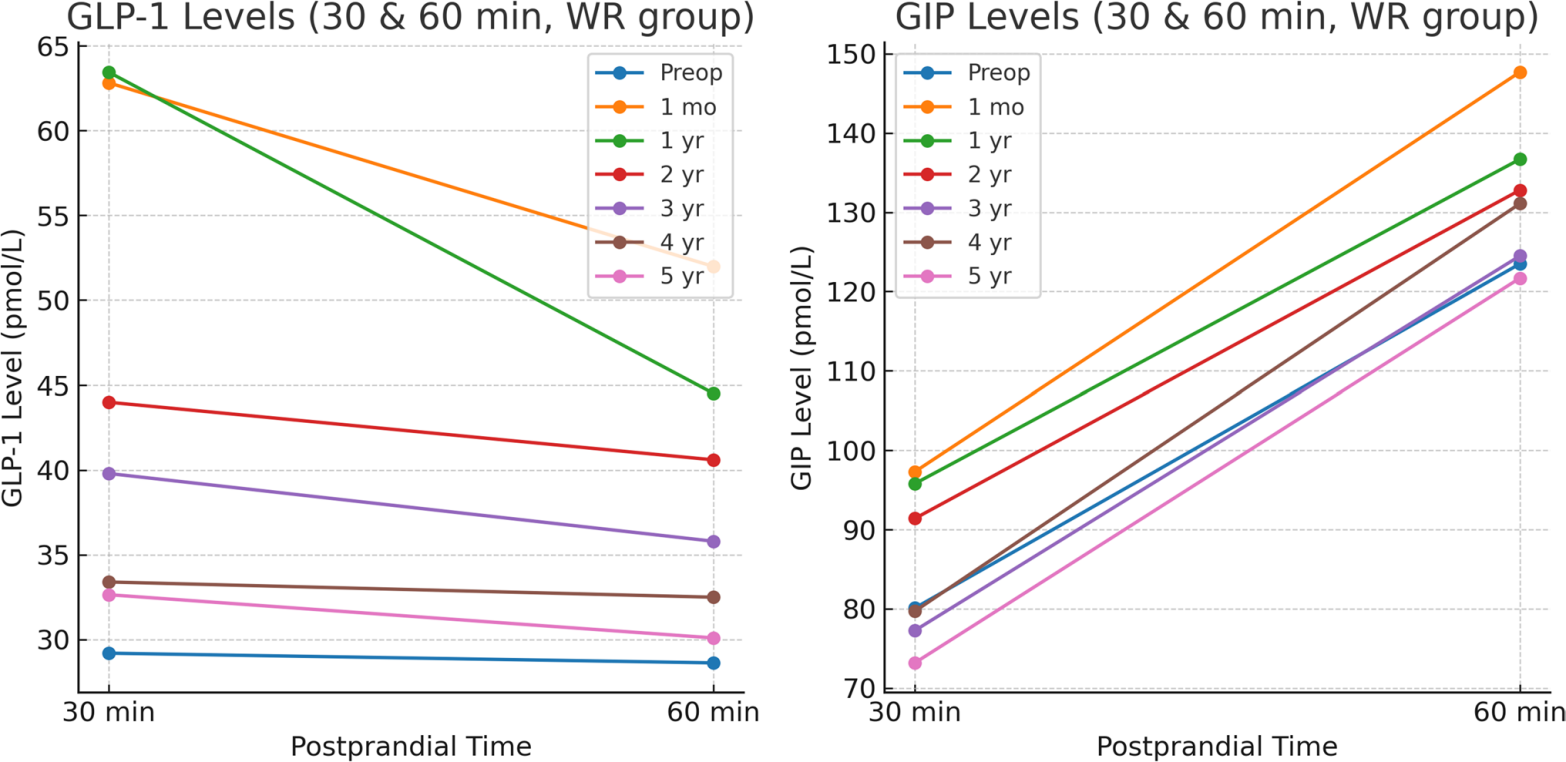
GLP-1 and GIP levels at 30 and 60 min postprandial in the clinically significant weight regain group

As shown in Table 5, the subgroup with clinically significant weight regain (≥ 10% weight regain from the nadir and %EWL < 50% at 18 months) demonstrated a similar early hormonal elevation in both GLP-1 and GIP, peaking at 1 month and 1 year postoperatively. However, the decline in hormone levels was more pronounced and occurred earlier than in the overall cohort. By year three, GLP-1 and GIP levels had substantially decreased, with some time points showing non-significant changes from baseline by year five. These patterns suggest that early attenuation of GLP-1 and GIP responses correlates with WR. However, the observed differences were modest, indicating only a potential association rather than a definitive predictive role for gut hormone trajectories in long-term outcomes (Figs. 5 and 6).

**Table 5:** GLP-1 and GIP levels in the clinically significant weight regain subgroup of the studied patients

(*n* = 62)	Fasting		30 min		60 min	120 min
GLP1 level (pmol/L)						
Preoperative	24.2 ± 8.24		29.2 ± 12.26		28.63 ± 12.31	23.6 ± 8.4
1 month	30.1 ± 8.05		62.82 ± 14.24		51.98 ± 13.23	40.8 ± 9.54
*P* value	** < 0.001**		** < 0.001**		** < 0.001**	** < 0.001**
Mean difference (95% CI)	5.92 (4.53: 7.31)		33.58 (31.31: 35.85)		23.35 (21.17: 25.54)	17.23 (15.69: 18.76)
1 year	34 ± 8.07		63.45 ± 14.38		44.5 ± 13.35	34.6 ± 10.27
*P* value	** < 0.001**		** < 0.001**		** < 0.001**	** < 0.001**
Mean difference (95% CI)	9.85 (8.46: 11.25)		34.21 (31.92: 36.49)		15.82 (13.63: 18.02)	11.03 (9.43: 12.64)
2 years	31.16 ± 8.33		44 ± 15.63		40.6 ± 13.24	30.9 ± 10.11
*P* value	** < 0.001**		** < 0.001**		** < 0.001**	** < 0.001**
Mean difference (95% CI)	7 (5.58: 8.42)		14.71 (12.31: 17.11)		11.95 (9.77: 14.14)	7.31 (5.72: 8.9)
3 years	30 ± 8.91		39.8 ± 14.16		35.8 ± 13.87	29.5 ± 10.31
*P* value	** < 0.001**		** < 0.001**		** < 0.001**	** < 0.001**
Mean difference (95% CI)	5.84 (4.37: 7.31)		10.58 (8.32: 12.85)		7.19 (4.95: 9.44)	5.94 (4.33: 7.54)
4 years	27.2 ± 8.02		33.4 ± 12.84		32.5 ± 13.3	26.3 ± 9.12
*P* value	** < 0.001**		** < 0.001**		** < 0.001**	** < 0.001**
Mean difference (95% CI)	3.05 (1.66: 4.44)		4.16 (2.02: 6.31)		3.82 (1.63: 6.01)	2.71 (1.21: 4.21)
5 years	24.8 ± 8.56		32.65 ± 13.11		30.1 ± 12.86	24.5 ± 8.83
*P* value	0.382		**0.006**		0.168	0.239
Mean difference (95% CI)	0.61 (−0.82: 2.05)		3.4 (1.23: 5.57)		1.44 (−0.72: 3.59)	0.94 (−0.54: 2.41)
GIP level (pmol/L)						
Preoperative		12.84 ± 4.55		80.1 ± 21.09	123.5 ± 25.15	62.9 ± 19
1 month		18.2 ± 5.2		97.3 ± 22.4	147.7 ± 26.21	75.4 ± 19.02
*P* value		** < 0.001**		** < 0.001**	** < 0.001**	** < 0.001**
Mean difference (95% CI)		5.32 (4.49: 6.16)		17.23 (13.51: 20.95)	24.23 (19.83: 28.62)	12.53 (9.28: 15.78)
1 year		21.3 ± 4.74		95.8 ± 21.78	136.7 ± 24.15	82.9 ± 18.95
*P* value		** < 0.001**		** < 0.001**	** < 0.001**	** < 0.001**
Mean difference (95% CI)		8.44 (7.64: 9.23)		15.69 (12.03: 19.36)	13.27 (9.06: 17.49)	20 (16.75: 23.25)
2 years		18.4 ± 4.51		91.4 ± 21.8	132.8 ± 24.63	71.8 ± 20.34
*P* value		** < 0.001**		** < 0.001**	** < 0.001**	** < 0.001**
Mean difference (95% CI)		5.55 (4.77: 6.32)		11.26 (7.59: 14.92)	9.29 (5.03: 13.55)	8.95 (5.59: 12.32)
3 years		16.9 ± 4.49		77.26 ± 22.02	124.5 ± 25.23	68.4 ± 19.05
*P* value		** < 0.001**		**0.003**	0.404	** < 0.001**
Mean difference (95% CI)		4.03 (3.26: 4.81)		−2.84 (−6.52: 0.85)	1.03 (−3.27: 5.34)	5.53 (2.28: 8.79)
4 years		15.4 ± 4.39		79.7 ± 21.53	131.1 ± 25.23	66.7 ± 18.91
*P* value		** < 0.001**		0.742	** < 0.001**	** < 0.001**
Mean difference (95% CI)		2.55 (1.78: 3.31)		−0.35 (−4: 3.29)	7.68 (3.33: 12.03)	3.81 (0.57: 7.05)
5 years		13.8 ± 4.31		73.18 ± 21.47	121.7 ± 25.53	69.5 ± 19.09
*P* value		0.088		** < 0.001**	0.251	** < 0.001**
Mean difference (95% CI)		0.92 (0.16: 1.68)		−6.92 (−10.56: −3.28)	−1.76 (−6.09: 2.57)	6.6 (3.34: 9.85)

Data are presented as mean ± SD. *CI* Confidence interval

*P*-values and 95% CIs reflect comparisons to baseline hormone levels at each time point within the weight regain group

Despite the persistent significant increase in endogenous GLP-1 in the second year, this group showed > 10% WR. Other factors other than the endogenous GLP-1 play the main role in the WR. The endogenous GIP levels showed no significant role in the WR.

### Effect of Semaglutide in Patients with the Clinically Significant Weight Regain (WR)

There were no significant differences in age, sex, or baseline comorbidities between WR and non-WR groups. By the time of follow-up, all patients had reached adulthood (> 18 years). Semaglutide therapy was initiated at the start of the third postoperative year in these patients, administered as weekly subcutaneous injections (0.5–1.0 mg) over a 12-month period, followed by a gradual tapering and a subsequent observation period of at least one year.

Table 6 highlights the impact of semaglutide on weight, BMI, %TWL, %EWL, and gastric volume among patients who experienced clinically significant weight regain following LSG. Notably, weight and BMI increased significantly between years 1 and 2, coinciding with reduced %TWL from 33.2% to 18% and %EWL from 63.3% to 34.1%. Following the introduction of semaglutide at beginning of year 3, patients demonstrated a meaningful recovery in weight loss metrics, with %EWL rising to 68% at year 3 and remaining significantly improved through year 4 (64.1%) and year 5 (58.3%) and %TWL rising to 35.56% at year 3 and remaining significantly improved through year 4 (33.5%) and year 5 (30.5%). While a slight decline in %EWL was observed after the cessation of therapy, the overall trajectory indicates a beneficial, though partially transient, effect of semaglutide. Despite continued increases in remnant gastric volume postoperatively, semaglutide effectively mitigated the adverse metabolic consequences of anatomical changes. These findings support the role of GLP-1 receptor agonists as a rescue therapy in adolescents with post-LSG weight regain, particularly when hormonal attenuation and anatomical dilation coexist.

**Table 6:** Weight, BMI, % TWL, % EWL and gastric volume in the clinically significant weight regain patients

(*n* = 62)		*P* value	Mean difference (95% CI)
Weight (kg)			
1 year	89.8 ± 12.69		
2 years	109.9 ± 11.41	** < 0.001**	20.11 (18.05: 22.18)
Weight (kg) after Semaglutide			
3 years	86.8 ± 15.27	**0.006**	−3.05 (−5.45: −0.65)
4 years	89.4 ± 15.3	0.756	−0.37 (−2.77: 2.03)
5 years	93.5 ± 15.59	**0.005**	3.71 (1.28: 6.14)
BMI (kg/m^2^)			
1 year	33.5 ± 6.96		
2 years	40.9 ± 6.97	** < 0.001**	7.42 (6.23: 8.62)
BMI (kg/m^2^) after Semaglutide			
3 years	32.4 ± 7.82	**0.009**	−1.1 (−2.37: 0.17)
4 years	33.4 ± 7.94	0.858	−0.08 (−1.36: 1.2)
5 years	34.9 ± 8.02	**0.004**	1.41 (0.12: 2.69)
TWL (%)			
1 year	33.2 ± 6.19		
2 years	18 ± 5.47	** < 0.001**	−15.18 (−16.18: −14.18)
TWL (%) after Semaglutide			
3 years	35.56 ± 8.3	**0.005**	2.37 (1.12: 3.62)
4 years	33.5 ± 8.46	0.711	0.34 (−0.93: 1.61)
5 years	30.5 ± 8.75	**0.006**	−2.73 (−4.03: −1.43)
EWL (%)			
1 year	63.3 ± 17.09		
2 years	34.1 ± 11.74	** < 0.001**	−29.28 (−31.79: −26.78)
EWL (%) after Semaglutide			
3 years	68 ± 20.96	**0.005**	4.66 (1.39: 7.93)
4 years	64.1 ± 20.88	0.660	0.8 (−2.47: 4.06)
5 years	58.3 ± 20.84	**0.01**	−5.08 (−8.34: −1.82)
Gastric volume (ml)			
1 year	123.6 ± 19.52		
2 years	177.5 ± 18.32	** < 0.001**	53.89 (50.65: 57.12)
Gastric volume (ml) after Semaglutide			
3 years	188.2 ± 19.49	** < 0.001**	64.58 (61.24: 67.92)
4 years	196.6 ± 19.3	** < 0.001**	73 (69.68: 76.32)
5 years	222.1 ± 18.12	** < 0.001**	98.42 (95.2: 101.64)

Data are presented as mean ± SD. *TWL* Total weight loss, *EWL* Excess weight loss, *CI* Confidence interval

We conducted a comparative analysis between patients with clinically significant weight regain treated with semaglutide (n = 62) and those without weight regain (n = 202) over a five-year period. Non-WR patients exhibited significantly higher mean %EWL at years 4 and 5, smaller remnant gastric volumes, and more sustained postprandial GLP-1 and GIP responses at year 3 (all p < 0.05). The between-group 95% confidence intervals further confirmed these differences, indicating meaningful separation in outcome trajectories. In contrast, WR patients, despite showing semaglutide-induced improvements, experienced earlier attenuation of incretin responses and progressive anatomical dilation. These findings underscore semaglutide’s role as a rescue intervention rather than a preventive measure and support the exploration of early hormonal monitoring and anatomical surveillance as possible tools for identifying high-risk individuals (Table 7), acknowledging that further validation is warranted.

**Table 7:** Comparative metrics between the clinically significant WR patients (with Semaglutide) and non-WR patients

Parameter	WR + Semaglutide (*n* = 62)	Non-WR (*n* = 202)	*P*-value	95% CI (Between Groups)
Mean %TWL at 3 years	35.56 ± 8.3	38.69 ± 6.2	**0.002**	−4.39 to −1.88
Mean %TWL at 4 years	33.5 ± 8.46	36.41 ± 6.3	**0.004**	−4.15 to −1.63
Mean %TWL at 5 years	30.5 ± 8.75	35 ± 6.73	** < 0.001**	−5.84 to −3.25
Mean %EWL at 3 years	68.0 ± 20.96	71.2 ± 18.77	0.091	−3.2 to 9.6
Mean %EWL at 4 years	64.1 ± 20.88	69.3 ± 17.56	**0.043**	−0.2 to 10.4
Mean %EWL at 5 years	58.3 ± 20.84	66.4 ± 16.92	**0.017**	1.0 to 12.1
Postprandial GLP-1 (30 min) at 3 years (pmol/L)	39.8 ± 14.16	47.5 ± 13.20	**0.004**	3.1 to 11.4
Postprandial GIP (30 min) at 3 years (pmol/L)	77.26 ± 22.02	84.6 ± 20.35	**0.031**	1.1 to 13.6
Remnant Gastric Volume at 5 years (mL)	222.1 ± 18.12	192.3 ± 17.84	** < 0.001**	−36.8 to −23.1

Data are presented as mean ± SD, *TWL* Total weight loss, *EWL* Excess weight loss, *CI* Confidence interval

**Fig. 7:**
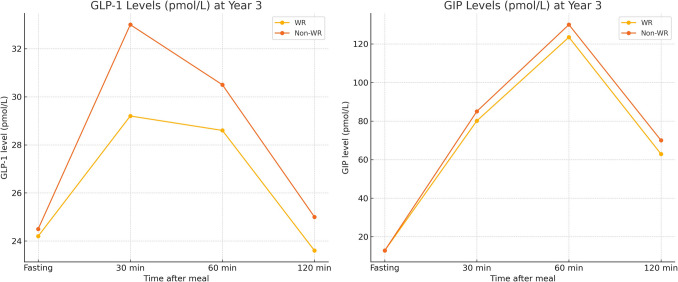
Comparative GLP-1 and GIP levels in patients with clinically significant weight regain (WR) vs. non-weight regain (non-WR) at year 3 post-sleeve gastrectomy. The WR group exhibited attenuated postprandial hormonal responses, particularly at 30 and 60 min after meal ingestion

To further delineate hormonal patterns, we compared postprandial GLP-1 and GIP levels between WR and non-WR groups. As shown in Table 7 and Fig. 7, WR patients exhibited significantly lower postprandial GLP-1 and GIP levels at year 3 compared to non-WR patients (p = 0.004 and 0.031, respectively), suggesting earlier hormonal attenuation. These differences highlight that hormonal monitoring may have potential to anticipate weight trajectory post-LSG, but the predictive value remains to be validated.

## Discussion

Laparoscopic sleeve gastrectomy (LSG) remains an effective treatment for adolescent obesity, offering substantial and sustained weight loss (WL) and metabolic improvement. This study reaffirms these benefits, with a marked reduction in BMI and comorbidities over five years. Notably, it provides novel insights into the longitudinal behavior of gut hormones (GLP-1 and GIP) and their potential link to weight regain (WR) and semaglutide responsiveness.

### Weight Loss and Regain Patterns

Our data demonstrates significant and sustained weight reductions following LSG in adolescents with morbid obesity. The observed weight loss and BMI at two years post-surgery, followed by partial WR, aligns with patterns documented in the literature. This mirrors findings from Al Sabah et al. [21], who reported peak %EWL of 82.66% at 18 months in adolescents undergoing SG, with sustained but diminishing weight reduction thereafter. Başaran et al. [18], noted that metabolic adaptations following BS can contribute to WR, potentially involving alterations in energy expenditure and fat oxidation. Our findings, which indicate a gradual decline in %EWL and hormonal responses over five years, align with the understanding that long-term outcomes post-LSG can be variable. This is further supported by studies like that of Shehata et al. (2025), who, in their seven-year follow-up of adolescent LSG patients, observed sustained benefits but also noted modest weight regain in some cases, emphasizing the ongoing need for individualized care and potentially, adjunctive strategies to maintain optimal results [11]. However, despite this partial WR, our cohort maintained substantial improvements compared to baseline, with an %EWL of 63.3% at five years. This finding underscores the efficacy of LSG as an intervention for morbid obesity in adolescents, while highlighting the need for adjunctive strategies to optimize long-term outcomes.

### Hormonal Adaptations and Weight Trajectory

LSG removes ~ 80% of the stomach, including the fundus (a major site of ghrelin production and gastric reservoir function). This dramatically reduces gastric capacity and speeds up gastric emptying, so nutrients reach the distal small intestine (ileum) much faster [22]. The L-cells in the ileum and colon are the primary source of GLP-1, and they are activated by direct contact with nutrients especially fats and carbohydrates [23].

Despite partial WR, the sustained improvements in glycemic parameters observed in our cohort suggest that the metabolic benefits of LSG extend beyond WL effects. The progressive reduction in HbA1c from 7.12% preoperatively to 5.36% at five years mirrors findings from Casella et al. [24], who documented significant improvements in insulin sensitivity following SG attributed primarily to enhanced GLP-1 secretion rather than WL alone. Similarly, Nannipieri et al. [25], reported that improved β-cell glucose sensitivity after BS was positively associated with GLP-1 response. These findings collectively support the concept that SG induces favorable alterations in glucose metabolism through weight-independent mechanisms.

The marked reduction in DM, hypertension, and dyslipidemia prevalence observed in our cohort suggests comprehensive improvements in metabolic health following LSG in adolescents. The progressive nature of comorbidity resolution, with continued improvements beyond the period of maximum WL, indicates that mechanisms beyond adiposity reduction contribute to these benefits. Our findings align with those of Vigneshwaran et al. [26], who reported complete remission of T2D in 50% of patients following LSG, attributing this effect to elevated postprandial GLP-1 levels and subsequent enhancement of insulin secretion. Shehata et al. [27] further confirmed the metabolic benefits of LSG, showing significant remission rates of T2D (66.7%) and GERD (40%) at 5 years post-surgery in a female reproductive cohort. The multifaceted actions of incretin hormones, as described by Seino et al. [12], likely contribute to these improvements, with GLP-1 promoting β-cell proliferation, inhibiting apoptosis, and suppressing glucagon secretion. The resolution of comorbidities in our adolescent cohort mirrors the findings of Al Sabah et al. [21], who reported significant improvements in comorbidities following SG in youth.

Our study revealed significant alterations in fasting and postprandial GLP-1 and GIP levels following LSG, with peak enhancements occurring at one year post-surgery, followed by gradual attenuation.

These patterns align with findings from Shehata et al. [10], who documented marked increases in GLP-1 levels within the first 6 months after SG, regardless of surgical technique, with diminishing effects thereafter. Similarly, Min et al. [28], reported significant elevations in post-glucose GLP-1 levels at 1 and 6 months following LSG but a return to baseline by 4th year.

The robust postprandial GLP-1 response observed in our cohort, particularly at one year post-surgery, is consistent with Dimitriadis et al. [29], who found significantly enhanced postprandial GLP-1 release at 60 and 120 min after meals at both 6 and 12 months post-LSG. This exaggerated meal-stimulated GLP-1 release likely contributes to improved satiety and metabolic benefits. The parallel increases in GIP levels observed in our cohort, followed by gradual normalization, correspond with findings from Mccarty et al. [13], who reported significant increases in postprandial GLP-1 after SG but no significant change in fasting GIP levels in their meta-analysis.

While we used discrete postprandial time-point analyses to illustrate hormonal trends over time, we acknowledge that calculation of the area under the curve (AUC) may offer a more integrated measure of hormonal exposure and response. This approach could simplify comparisons and better reflect the physiological impact of incretin secretion. Future studies should incorporate AUC-based assessments to better quantify hormonal dynamics postoperatively and their relationship to clinical outcomes.

GLP-1 attenuates over time due to the gut adapts to new nutrient flow patterns. L-cell stimulation becomes less exaggerated as the intestines accommodate to the faster nutrient exposure and vagal and enteric nervous system signaling may also adjust, moderating GLP-1 release [30]. Also, patients consume small, carbohydrate-rich meals in the early postoperative phase, which strongly stimulate GLP-1. Over time, as patients transition to more balanced or protein-heavy diets with fewer simple carbohydrates, GLP-1 response may diminish. As weight decreases and insulin sensitivity improves, the body may no longer require the same level of GLP-1 secretion to maintain glycemic control [31].

While our original cohort-level data suggested generalized hormonal attenuation over time, our subgroup analysis (Table 7) and comparative plots (Fig. 7) demonstrate that WR patients experience a more rapid decline in GLP-1 and GIP levels postoperatively. These differences were moderate, suggesting a possible association with WR risk; however, prospective studies are needed to assess their predictive utility. It is also important to note that while our findings suggest temporal associations between early hormonal attenuation and WR, these are based on descriptive comparisons rather than inferential correlation testing. We did not perform Pearson or Spearman correlation between hormone levels and weight outcomes. Therefore, our conclusions about potential predictive value remain exploratory and hypothesis-generating.

### Semaglutide and Pharmacologic Rescue

Semaglutide is a GLP-1 receptor agonist used primarily for weight management and type 2 diabetes control. It helps reduce appetite, improve glycemic control, and promote weight loss [32].

In patients experiencing clinically significant WR, our findings demonstrate substantial diminishment of %EWL from 63.3% at one year to 34.1% by year two. The introduction of Semaglutide in this subgroup resulted in partial recapture of WL, with %EWL increasing to 68% at three years, though this effect appeared to wane by year five. This response pattern aligns with findings from Başaran et al. and Vidmar et al., who demonstrated initial efficacy but variable long-term responsiveness to GLP-1 therapies [18, 33]. Although randomized trials are still ongoing in adolescents, recent observational studies suggest semaglutide can be safely initiated in post-pubertal patients over 18 years with prior bariatric surgery [33, 34].

In our cohort, the observed improvement in %EWL following semaglutide administration supports the mechanistic hypothesis that post-surgical weight regain may, in part, be driven by diminishing endogenous incretin responses, which can be partially reversed with pharmacological GLP-1 receptor agonist therapy.

It is important to note that we did not stratify WR patients based on baseline GLP-1 or GIP levels prior to semaglutide initiation. Therefore, while hormonal attenuation was observed in the WR group overall, we cannot conclude that patients with lower endogenous incretin levels derive greater benefit from GLP-1 receptor agonist therapy. The current data demonstrate semaglutide efficacy in a phenotypically defined WR cohort but do not establish a predictive link between hormone levels and treatment response. Future prospective studies are needed to explore whether baseline or dynamic incretin profiles can serve as biomarkers for responsiveness to adjunct pharmacotherapy.

Notably, semaglutide treatment in our patients was limited to a 12-month course initiated in the third postoperative year and was gradually tapered over 3 months thereafter. Therefore, the reduction in its effect observed by year five likely reflects the cessation of therapy rather than the development of pharmacologic tolerance or adaptation. These findings suggest that longer or repeated courses of GLP-1 agonist therapy may be needed to sustain benefits and align with observations by Davies et al. [34], who demonstrated that higher doses of Semaglutide (2.4 mg) produced superior weight reductions compared to lower doses in adults with obesity and T2D.

Patients experiencing significant WR in our cohort exhibited distinctive gut hormone trajectories characterized by initial robust increases in GLP-1 and GIP levels followed by earlier attenuation than those maintaining WL. By year five, fasting GLP-1 levels in these patients approximated preoperative values, with substantially reduced postprandial responses compared to the early postoperative period. Paradoxical WR despite elevated GLP1 in years 2,3 after SG. Similar to insulin resistance, some patients may develop resistance or diminished sensitivity to GLP-1 effects over time. Elevated GLP-1 levels may no longer translate to appetite suppression or improved metabolism. Also, other orexigenic (appetite-stimulating) hormones such as Ghrelin may increase or rebound, offsetting GLP-1 effects [18].

Importantly, hormone sampling in patients treated with semaglutide was conducted without a washout period. This may have influenced endogenous GLP-1 measurements due to receptor-mediated feedback inhibition, as previously described [12, 13]. Despite this, postprandial GLP-1 and GIP responses were still detectable, suggesting that hormonal secretion persists under treatment, albeit at attenuated levels. The effect on GIP was less pronounced, consistent with prior findings that GLP-1 receptor agonists exert minimal direct suppression of GIP secretion [18, 32]. These considerations should be accounted for in interpreting comparative hormonal data between WR and non-WR patients.

This pattern aligns with mechanistic insights from previous studies. Yang et al. [35], demonstrated that while LSG induces significant increases in fasting GLP-1 levels at 1, 3, 6, and 12 months postoperatively, these changes are less pronounced than those following RYGB. Similarly, Alamuddin et al. [36], reported only modest increases in GLP-1 following vertical SG compared to more marked elevations after RYGB. The temporal Association between attenuating incretin responses and WR in our cohort supports findings from Nosso et al. [37], though that study found that metabolic improvements were more closely linked to WL than to gastrointestinal hormone changes. The distinct hormonal profiles observed in our WR group suggest potential utility in monitoring postprandial gut hormone responses as biomarkers for identifying patients at risk for suboptimal long-term outcomes.

Current evidence on the relationship between attenuated post-prandial GLP-1 responses and weight regain is limited [38]. Some studies have reported that post-prandial GLP-1 is negatively correlated with WR whereas post-prandial GIP shows a positive correlation [39]. These findings underscore the need for prospective studies evaluating these relationships.

Our interpretation aligns with recent findings by Başaran et al. [18], who linked early hormonal attenuation post-SG to suboptimal weight maintenance, and by Nosso et al. [37], who demonstrated that hormonal responses can differentiate long-term metabolic outcomes.

### Predictors and Mechanisms of WR

Remnant gastric volume was the only consistent correlate of WR in our cohort. Larger volumes likely enable greater food intake and diminished satiety, undermining hormonal and mechanical restrictions. No significant differences were observed in age, sex, or baseline comorbidities. This supports prior findings that anatomical and physiological factors may be more predictive of WR than demographics alone [23, 40].

Additionally, the hormonal patterns seen in WR patients (early attenuation of GLP-1 and GIP response) may act as potential biomarkers of surgical durability. Monitoring these hormones postoperatively may allow for earlier detection of WR risk.

### Implications for Clinical Practice

Our findings suggest that integrating hormonal surveillance with individualized pharmacotherapy may enhance long-term care of adolescents post-LSG, but further research is required to establish the utility and cost-effectiveness of such strategies. Routine monitoring of GLP-1 and GIP could guide timely interventions, while early semaglutide use may mitigate WR in responsive individuals.

The findings of our study have significant implications for the management of metabolic obesity in adolescents. The identification predictable patterns in weight trajectory and gut hormone responses following LSG provides a foundation for developing tailored postoperative monitoring and intervention strategies. In particular, the successful implementation of GLP-1 receptor agonist therapy for post-surgical WR demonstrates the potential for pharmacological approaches to enhance the durability of surgical outcomes. As suggested by Shah et al. [41], GLP-1 receptor agonists represent a promising adjunct to BS in pediatric populations, with Semaglutide demonstrating superior efficacy in recent trials.

Additionally, the sustained improvements in glycemic control and comorbidity resolution observed in our cohort, despite partial WR, highlight the complex physiological effects of SG beyond simple restriction. As revealed by Essop et al. [42], while GLP-1 agonists are highly effective for managing T2D and supporting WL, BS remains superior in achieving long-term diabetes remission.

The observed waning of endogenous incretin enhancement over time following LSG, particularly in patients experiencing WR, suggests that individual variations may influence the durability of surgical outcomes in gut hormone adaptation. This finding aligns with the concepts presented by Farey et al. [40], who noted that LSG alters certain hormonal profiles to more closely resemble those of non-obese individuals.

### Comparison to Other Bariatric Modalities

Studies have shown that Roux-en-Y gastric bypass (RYGB) produces more sustained hormonal effects and greater remission of type 2 diabetes than LSG [35, 36]. However, the lower complication rate and simpler anatomy make LSG preferable in adolescents. Understanding the hormonal limitations of LSG may help narrow the outcome gap with RYGB.

## Limitations

Despite the strengths of this retrospective cohort study, including its comprehensive five-year follow-up and detailed hormonal profiling, several limitations must be acknowledged. First, the non-randomized, single-center design may limit the generalizability of findings to broader adolescent populations or different surgical practices. Second, the study was powered solely based on anticipated changes in GLP-1 levels and was not designed to detect correlations between GLP-1 or GIP and weight regain (WR), nor to evaluate the differential effects of semaglutide across hormonal subgroups. Third, no formal correlation analyses (e.g., Pearson or Spearman) were performed, and all observed associations between hormonal attenuation and WR are descriptive and exploratory in nature. Fourth, semaglutide was administered only to patients meeting strict criteria for clinically significant WR and aged ≥ 18 years, without randomization, introducing potential selection bias. Fifth, hormonal measurements were performed while patients remained on semaglutide, which may have influenced endogenous GLP-1 levels through feedback inhibition (in one sample). Additionally, the use of a fixed 300 kcal mixed meal for all patients may have been suboptimal for older or larger adolescents, potentially contributing to the attenuated incretin responses observed over time. Furthermore, the study did not assess important behavioral or psychological variables such as dietary adherence, physical activity, or mental health, which are known to impact weight trajectories. Finally, other relevant gut hormones, including ghrelin and peptide YY, were not measured, limiting the comprehensiveness of the hormonal assessment. Lastly, while the five-year follow-up provides valuable long-term insights, the durability of semaglutide effects and hormonal adaptations into adulthood remains uncertain and warrants further investigation.

## Conclusions

Laparoscopic sleeve gastrectomy (LSG) remains a highly effective intervention for adolescent obesity, resulting in substantial weight loss, improved glycemic control, and resolution of obesity-related comorbidities over five years. Our findings also demonstrate distinct patterns in incretin hormone responses, with early postoperative increases in GLP-1 and GIP that gradually declining over time (changes that may be associated to WR in a subset of patients).

Among those experiencing clinically significant WR, adjunct semaglutide therapy was associated with partial weight loss recovery, supporting its role as a valuable adjunct to surgical management in selected patients. Notably, adolescents with WR showed earlier attenuation in postprandial hormonal responses and larger remnant gastric volumes, indicating that anatomical and hormonal factors may contribute to suboptimal long-term outcomes.

## Clinical Implications

These findings raise the possibility that early differences in gut hormone trajectories (particularly attenuated GLP-1 and GIP responses) may be linked to WR risk following LSG in adolescents. While not yet definitive predictors, such patterns warrant further investigation and may support future strategies incorporating hormonal surveillance, anatomical assessment, and timely pharmacologic adjuncts (e.g., GLP-1 receptor agonists) to optimize surgical outcomes. However, as the current associations are descriptive and not based on correlation or causal inference, prospective randomized studies with formal correlation analyses, extended follow-up, and broader hormonal profiling are essential to validate these observations and guide individualized treatment approaches.

## Data Availability

No datasets were generated or analysed during the current study.
